# A New Pipeline for Designing Phage Cocktails Based on Phage-Bacteria Infection Networks

**DOI:** 10.3389/fmicb.2021.564532

**Published:** 2021-02-16

**Authors:** Felipe Molina, Alfredo Simancas, Manuel Ramírez, Rafael Tabla, Isidro Roa, José Emilio Rebollo

**Affiliations:** ^1^Genetics, Department of Biochemistry Molecular Biology and Genetics, University of Extremadura, Badajoz, Spain; ^2^Microbiology, Department of Biomedical Sciences, University of Extremadura, Badajoz, Spain; ^3^Dairy Department, Scientific and Technological Research Centre of Extremadura, Technological Institute of Food and Agriculture, Junta de Extremadura, Badajoz, Spain

**Keywords:** *Escherichia coli*, cheese, dairy industry, phage-host coevolution, phage-bacteria infection networks, phage cocktails, phage therapy

## Abstract

In recent years, the spread of antibiotic-resistant bacteria and efforts to preserve food microbiota have induced renewed interest in phage therapy. Phage cocktails, instead of a single phage, are commonly used as antibacterial agents since the hosts are unlikely to become resistant to several phages simultaneously. While the spectrum of activity might increase with cocktail complexity, excessive phages could produce side effects, such as the horizontal transfer of genes that augment the fitness of host strains, dysbiosis or high manufacturing costs. Therefore, cocktail formulation represents a compromise between achieving substantial reduction in the bacterial loads and restricting its complexity. Despite the abovementioned points, the observed bacterial load reduction does not increase significantly with the size of phage cocktails, indicating the requirement for a systematic approach to their design. In this work, the information provided by host range matrices was analyzed after building phage-bacteria infection networks (PBINs). To this end, we conducted a meta-analysis of 35 host range matrices, including recently published studies and new datasets comprising *Escherichia coli* strains isolated during ripening of artisanal raw milk cheese and virulent coliphages from ewes’ feces. The nestedness temperature, which reflects the host range hierarchy of the phages, was determined from bipartite host range matrices using heuristic (Nestedness Temperature Calculator) and genetic (BinMatNest) algorithms. The latter optimizes matrix packing, leading to lower temperatures, i.e., it simplifies the identification of the phages with the broadest host range. The structure of infection networks suggests that generalist phages (and not specialist phages) tend to succeed in infecting less susceptible bacteria. A new metric (Φ), which considers some properties of the host range matrices (fill, temperature, and number of bacteria), is proposed as an estimator of phage cocktail size. To identify the best candidates, agglomerative hierarchical clustering using Ward’s method was implemented. Finally, a cocktail was formulated for the biocontrol of cheese-isolated *E. coli*, reducing bacterial counts by five orders of magnitude.

## Introduction

Soon after their discovery more than a century ago, viruses of bacteria, known as bacteriophages or phages, were appreciated as potential antibacterial agents. Later, the advent of antibiotics eclipsed further development of “phage therapy” in Western countries (Sulakvelidze et al., 2001). However, the spread of multidrug-resistant bacteria has become a daunting challenge for public health in the twenty-first century. Additionally, the so-called healthy microbiota found in fermented foods pose a limit to the use of antibiotics in food microbiology. To tackle these issues, strictly lytic (virulent) phages can be used as antibacterial agents in clinical and veterinary contexts or for reducing bacterial loads in foods and crops (Gutiérrez et al., 2016; Abedon et al., 2017). Virulent bacteriophages are natural predators of bacteria and may yield the complete destruction of bacterial lineages, releasing offspring into the surrounding environment and producing rapid exponential proliferation. Moreover, the selective toxicity of phages avoids harming the useful microbiota in vertebrates and foods (Rea et al., 2011; De Paepe et al., 2014; Lugli et al., 2016). In the dairy industry, *E. coli*, usually originating from animal feces, can form biofilms on food processing surfaces (Fernández et al., 2017) and ruin cheese-making, causing early blowing and rendering a final product unsuitable for human consumption. Raw milk cheeses, particularly soft and semihard varieties, have been associated with pathogenic *E. coli* outbreaks (Altekruse et al., 1998), but dairy products cannot be treated with antibiotics since they inhibit the growth of lactic acid bacteria (Marcó et al., 2014). In contrast, bacteriophages are more specific and do not affect the organoleptic properties of cheese.

**FIGURE 1:**
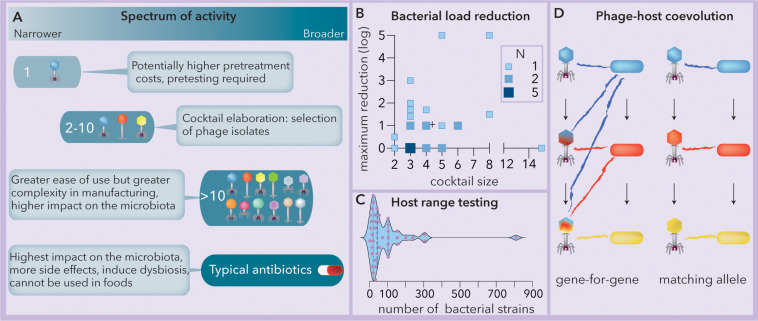
The spectrum of activity of phage cocktails relies on phage-host coevolution. **(A)** Summary of the spectra of activities, benefits and tradeoffs of phage-based formulations vs. typical commercial antibiotics (modified from Chan et al., 2013). **(B)** Observed bacterial load reduction vs. phage cocktail size (data extracted from Chan et al., 2013). The crosshair symbol (+) represents the center of the data. *N* = number of studies (Spearman *r* = 0.17, *p* = 0.387). **(C)** Responses to the question “how many strains do you actually use in testing host range?”; when the number is variable, only the maximum is considered. *N* = 40. Modified from Hyman (2019). The median and quartiles are represented by dashed and dotted lines, respectively. The data were collected using a survey at the 2017 Evergreen International Phage Meeting and from several people who work in phage-related companies. **(D)** Two modes of phage-host coevolution: gene-for-gene and matching allele correspond to generalist and specialist phage populations, respectively. Mutations are indicated by arrows and cross infection by colored lines. Ancestral (blue), intermediate (red) and newly evolved (yellow) bacterial lineages are represented.

Phage therapy can be implemented using either a single phage strain or a “cocktail” composed of a variable number of phages (Figure 1A). The use of a single lineage requires the identification of the phage with the broadest host range. However, while most plant and animal viruses present broad host ranges, phages face tradeoffs between their host range extension and their fitness (virulence and structural stability) in a particular niche (Duffy et al., 2006; Koskella and Meaden, 2013). In addition, bacteria can resist phage attack by mechanisms such as restriction-modification systems, adaptive immunity and spontaneous mutations (Labrie et al., 2010). Consequently, the host range of phages (spectrum of activity) tends to be narrow, often affecting subsets of strains within a single bacterial species (Hyman and Abedon, 2010). This narrow range can hinder the ability of single phage strains to impact bacterial proliferation (Malik et al., 2017). On the other hand, for some phages, the host range might be quite broad, spanning multiple bacterial genera (Balogh et al., 2010). Phage cocktails can be tailored by combining multiple isolates to broaden the spectrum of lysis, and later, they can be reformulated if resistance develops (Chan et al., 2013). The use of complex cocktails (more than 10 phages) is expected to increase the spectrum of activity as well as the production costs, the impact on the microbiota (dysbiosis) and the risk of horizontal transfer of toxins, antibiotic resistance or virulence genes (Penadés et al., 2015; Haaber et al., 2016; Colavecchio et al., 2017). Generally, a cocktail composed of between 2 and 10 phages represents the optimum between the two extremes (Figure 1A).

Although guidelines to compose phage cocktails have been proposed to comply with quality and safety requirements (Merabishvili et al., 2017), several pieces of evidence strongly indicate the convenience of developing a pipeline to design them. For instance, a comparison of 31 studies (this work, data extracted from Chan et al., 2013) reveals that the observed bacterial load reduction does not increase significantly with the size of the phage cocktail (Figure 1B). The high rate of spontaneous mutation produces rapid phage-bacterial coevolution that makes it difficult to predict the success of phage cocktails (Torres-Barceló, 2018). Although the structure of host range matrices (Cairns et al., 2009) relies on the coevolutionary dynamics of phages and hosts (Koskella and Brockhurst, 2014) and may help to reveal the minimum effective cocktail size, in a recent survey (Hyman, 2019), very little agreement was found for the number of bacteria needed (most answers fluctuated between 20 and 100) for host range determination (Figure 1C).

In this work, we attempt to answer two questions that arise when formulating a phage cocktail: how many and which isolates should constitute the cocktail? To this end, we propose that the phage-bacteria infection network (PBIN) properties should be considered for the formulation of phage cocktails. Specifically, we look at the fill (fraction of successful infections), nestedness temperature, size (measured as the number of phages multiplied by the number of bacteria) and symmetry (number of phages vs. number of bacteria). Remarkably, PBINs are built from host range matrices, and their structure depends on the phage-host coevolution pattern (Weitz et al., 2013). Two main alternative mutation and selection models explain phage-host coevolution (Figure 1D). According to the gene-for-gene model, some phage mutations increase infectivity, favoring host range expansion, whereas in matching allele dynamics, mutations usually lead to specialization and the loss of infectivity against ancestral host strains. Conversely, bacterial mutations modifying the structure of surface phage receptors and conferring resistance to recently evolved phages might compromise resistance to ancestral phages (matching allele) or not (gene-for-gene) (Agrawal and Lively, 2003). Here, two algorithms that build PBINs from host range matrices, one genetic (Rodríguez-Gironés and Santamaría, 2006) and one heuristic (Atmar and Patterson, 1993), were compared. Subsequently, a meta-analysis, including 32 recently published assays and 3 host range matrices composed of *E*. *coli* strains from artisanal raw milk cheese and coliphages isolated from sheep feces, was performed to evaluate experimental and theoretical PBINs. Subsequently, Φ, a new estimator of phage cocktail size, is presented, and agglomerative hierarchical clustering was used to identify the best candidates for biocontrol of cheese-isolated *E. coli*. The resulting cocktail reduced bacterial counts by five orders of magnitude.

## Results

### An Overview of the Pipeline: Building Phage-Bacteria Infection Networks From Host Range Matrices to Design Phage Cocktails

**FIGURE 2:**
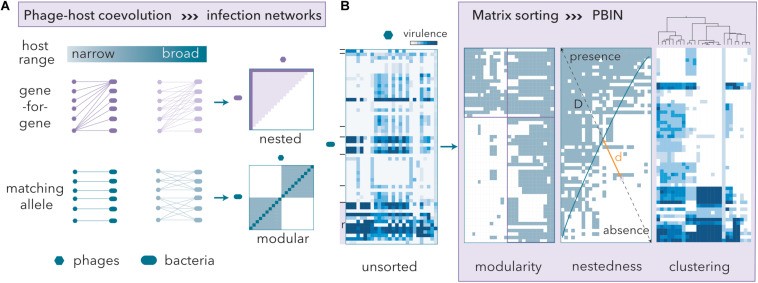
Phage-host coevolution determines phage-bacteria infection networks (PBINs). **(A)** Gene-for-gene coevolution entails nested PBINs, whereas matching allele coevolution favors modularity. Extreme modularity leads to non-inclusive sets of phage-bacteria interactions, i.e., one-to-one patterns (dark blue line in the modular panel). As the infectivity of the phages increases, so does the matrix fill (the dark magenta line in the nested panel represents a 10% fill fully nested matrix, whereas the light area corresponds to a 50% fill fully nested matrix). **(B)** Host range matrices may be sorted into different types of PBINs by using different reshuffling algorithms. A quantitative unsorted host range matrix comprising 44 artisanal cheese-isolated *E. coli* strains, 22 reference (r) *E. coli* strains and 26 coliphages isolated from sheep feces is shown. The cheese-isolated *E. coli* strains were grouped (black lines) by their whole-cell protein profiles (data not shown). Three different methods to build a network from the host range matrix are shown. Information about the virulence of the phages is lost when a quantitative matrix is transformed into a bipartite (presence/absence) form. Modularity was assessed by the LP-BRIM algorithm. Nestedness algorithms reorder host range data and estimate the deviation from a perfectly nested matrix (temperature) by computing the unexpected presence/absence values and measuring the relative distances (d/D) to the isocline of perfect order (blue line). The temperature of the matrix is the normalized sum of distances. Agglomerative hierarchical clustering by Ward’s method separated groups of phages with similar host range profiles.

The order of rows and columns in binary host range matrices can be permuted, revealing host-phage coevolution, without changing the underlying network structure (Figure 2A). The gene-for-gene model renders nested PBINs, whereas the matching allele model produces modular patterns (Weitz et al., 2013). Nestedness correlates with host range hierarchy, and therefore, high nestedness simplifies the identification of candidate phages for therapy. In a perfectly nested PBIN, only one phage is required to eradicate all bacteria. Conversely, modular PBINs might require high numbers of phage isolates to control the bacterial communities since a phage isolate from each module should be incorporated into the cocktail. Additionally, the size of the cocktail should inversely correlate with the fill of a matrix, which in turn reflects the infectivity of phage populations.

To design a phage cocktail, we will use a previously obtained quantitative host range matrix (Molina et al., 2020) comprising reference *E. coli* strains (Table 1) and isolates from goat and ewe raw milk cheeses and coliphages isolated from sheep feces (Figure 2B). To evaluate both modularity and nestedness, the heatmap was transformed into bipartite values (lysis/no lysis). As expected (see Discussion), low modularity was obtained (Barber’s Q = 0.197). To measure the nestedness, we compared two algorithms (see below) that reorder host range matrices and computed the deviation (temperature) from similarly filled matrices that are perfectly nested (Figure 2B). The nestedness temperature is normalized in such a way that it will always be a positive number smaller than or equal to 100. The size of phage cocktails was determined after considering the structure of the nested PBINs, and the phage isolates were selected after hierarchical clustering of the original quantitative matrix (Figure 2B).

**TABLE 1:** Bacterial strains used as references in this study.

Organism	Source and reference
*Citrobacter freundii*	CECT 7464
*Citrobacter youngae*	CECT 5335
*Enterobacter aerogenes*	CECT 648
*Escherichia coli*	
K-12 (MG1655)	This laboratory (Molina et al., 1998), ATCC 700926
MG1655 λ+	This laboratory
B (Luria)	CECT 4201
B/r	CECT 105
Bi	CECT 4537
BW6164	CGSC 6759
C (Sinsheimer)	ATCC 13706
W1 (Waskman)	CECT 99
W2 (Stoke)	CECT 727
C600	Gift from Dr. Rouviere-Yaniv, ATCC 23724
GY752	This laboratory (Sommer et al., 1998)
VIP45 λ+	Gift from Dr. Miguel Vicente
*Hafnia alvei*	CECT 158
*Klebsiella pneumoniae*	CECT 143
*Lactobacillus acidophilus*	CECT 903
*Lactobacillus casei*	CECT 475
*Lactococcus lactis ssp. Lactis*	CECT 185
*Salmonella typhimurium*	CECT 722
*Serratia marcescens*	CECT 846
*Shigella boydii*	CECT 583
*Shigella flexneri* 2a	CECT 585
*Shigella flexneri* 2b	CECT 4804
*Shigella sonnei*	CECT 4887
*Yersinia enterocolitica*	CECT 4315

*ATCC, American Type Culture Collection (United States); CECT, Colección Española de Cultivos Tipo (Spain); CGSC, Coli Genetic Stock Center (United States).*

### A Genetic Algorithm Optimizing the Nestedness of Host Range Matrices

**FIGURE 3:**
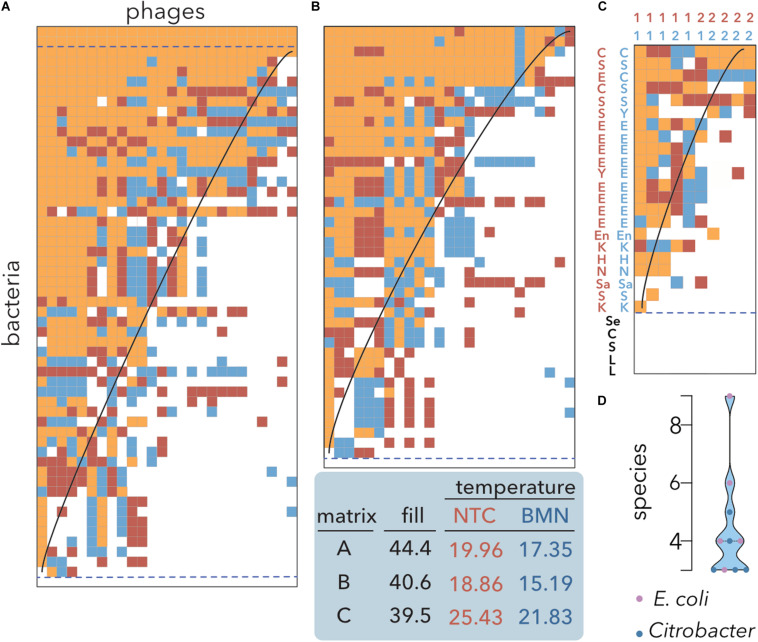
Nestedness analysis of *E. coli* isolates and coliphages. Data sorting and temperature calculation were carried out using the NTC (red) and BMN (blue) algorithms, and cells occupied by both methods are indicated by orange shading. The isoclines of perfect order, i.e., the curves separating filled and empty table cells in a perfectly nested matrix of the same size and fill, are overlaid. Matrices’ fills after packing and temperatures are indicated. Empty and multiple fully filled rows (dashed lines) were not considered to avoid redundancy for temperature estimation. **(A)** Lysis profiles of 26 coliphages isolated from sheep feces on 44 *E*. *coli* isolates from artisanal cheese and 12 reference *E. coli* strains (Table 1). **(B)** Reference *E*. *coli* strains were removed from the dataset. **(C)** A host range matrix comprising coliphages, *Citrobacter* phages and diverse bacterial strains was sorted. 1: coliphages; 2: *Citrobacter* phages; C: *Citrobacter*; E: *E*. *coli*. H: *Hafnia*; K: *Klebsiella*; L: *Lactobacillus* and *Lactococcus*; Sa: *Salmonella*; En: *Enterobacter*; Se: *Serratia*; S: *Shigella*; Y: *Yersinia* (Table 2) **(D)** Distribution of the number of species infected by the phages shown in **(C)**. Coliphages (purple) and *Citrobacter* phages (blue) are distinguished. The median and quartiles are represented by dashed lines.

To calculate the nestedness temperature of host range matrices, heuristic algorithms, such as the NTC, are frequently employed (Poullain et al., 2008; Flores et al., 2011; Weitz et al., 2013). However, to the best of our knowledge, there is no previous work using genetic algorithms, such as BMN, to study PBINs. Since BMN was developed to optimize the packing of matrices (Rodríguez-Gironés and Santamaría, 2006), we decided to compare both metrics to reorder rows and columns of host range matrices and estimate the nestedness temperatures (Figure 3).

To assess whether there are tradeoffs in which locally adapted phages suffer fitness costs in infecting other hosts, the original bipartite input matrix (Figure 2B) and the matrix without reference E. coli strains were compared (Figure 3A vs. Figure 3B). Both algorithms produced highly nested PBINs, but BMN rendered lower temperatures, i.e., increased nestedness. Interestingly, only one cheese-isolated *E. coli* strain was resistant to all the phages (Figures 3A,B bottom line), but there was not a single phage isolate able to infect the remaining 43 *E. coli* strains. The inclusion of reference bacterial lineages increased both the fill and the nestedness temperature of the matrix but did not change its structure significantly. Accordingly, three reference E. coli strains were lysed by all the phages (Figure 3A, three topmost rows), whereas none of the cheese isolates were infected by all the phages. These results suggest that local adaptation of phages does not compromise infection of other bacterial lineages, i.e., evolution favors generalist phages (see section “Discussion”). As expected, when a more heterogeneous although smaller matrix is packed (Figure 3C), the fill decreases but the nestedness temperature increases. Coliphages depicted a broader host range than *Citrobacter* phages isolated from sewage when different enterobacteria species were used as hosts. Notably, all the phages infected different species, ranging between 3 and 9 different species, although none predated either all the strains of the preferential host species or the control lactic acid bacteria (Figure 3D). Overall, these results demonstrate the superior packing of BMN, yielding consistently lower nestedness temperatures than the NTC.

**FIGURE 4:**
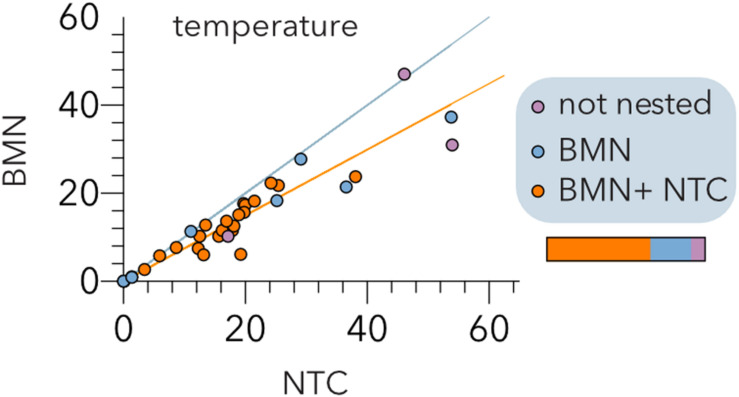
Comparison of the BinMatNest (BMN) and Nestedness Temperature Calculator (NTC) algorithms. The temperatures of the experimental host range matrices (Table 2) after sorting by each algorithm are indicated. The bisecting (cyan) and regression (orange) lines are indicated. The color coding indicates whether the matrix nesting is statistically significant (*p* < 0.05) by both algorithms (orange), BMN (blue) or none (purple) of the algorithms. The horizontal bar represents the relative abundance of each type of matrix.

To further validate these results, we conducted a meta-analysis (Table 2) assembling data from 32 studies of host-phage infection assays representing the cumulative study of 1,210 bacterial isolates, 703 phage isolates, and 33,428 separate attempts to infect a bacterial host with a phage strain. This analysis includes samples from different sources, such as plants, livestock, the dairy industry, sewage, seafood, clinical isolates and laboratory collection strains. Although most (65.7%) host range matrices were significantly nested by both algorithms (Figure 4), BMN consistently produced lower nestedness temperatures than the NTC, i.e., enhanced the packing of the matrices. Additionally, as the nestedness decreased, the difference between the temperature calculated by both algorithms increased. Only 3 (8.6%) PBINs were not statistically nested by the BMN algorithm (Table 2).

**TABLE 2:** Meta-analysis of experimental phage-bacteria infection networks and estimation of phage cocktail size.

References	Bacteria		Phages		Fill	Temp.^a^	Nested^b^	Φ^c^
Hong et al. (2013)	Laboratory	7	Sewage	3	71.4	0	+	1
Shende et al. (2017)	Sewage water	8	Manure	5	55	1.1	+	1
Liao et al. (2019)	Laboratory	17	Non-fecal compost	4	42.6	21.5	+	3
Krasowska et al. (2015)	Laboratory, soil	19	Soil	4	68.4	6.2	+	1
Hwang et al. (2009)	Poultry, laboratory	16	Poultry, sewage, soil	6	31.3	11.4	+	2
Kwiatek et al. (2015)	Clinical	20	Sewage	5	52	27.8	+	3
Hammerl et al. (2016)	Laboratory	36	Prophage induction	3	64.8	47.1	−	4
Xie et al. (2016)	Laboratory	12	Manure, cattle feed, water, soil	10	51.7	17.7	+	2
Magare et al. (2017)	Air	5	Air	25	15.2	31	−	3
Álvarez et al. (2019)	Potato, laboratory	42	River water	3	90.4	0	+	1
Pereira et al. (2016)	Food, water, laboratory	42	Sewage	3	62.7	0	+	1
Yu et al. (2016)	Kiwifruit	31	Soil	5	25.7	18.3	+	4
Dias et al. (2013)	Livestock	20	Sewage	10	92	10.3	−	2
Alič et al. (2017)	Orchid, wastewater	55	Orchid, wastewater	4	25	7.5	+	4
This work (Figure 3C)	Dairy, laboratory	26	Manure, sewage, laboratory	10	33.4	21.8	+	4
Salifu et al. (2013)	Equine	27	Soil	10	57.4	15.7	+	3
Arachchi et al. (2013)	Seafood	50	Laboratory	6	84.7	1	+	1
Oh et al. (2017)	Laboratory	27	Fermented food, soil	12	44.4	22.3	+	3
Wandro et al. (2019)	Human feces	15	Sewage	22	72.4	5.8	+	1
Gunathilaka et al. (2017)	Laboratory	12	Sewage	29	49.4	10.2	+	2
Jurczak-Kurek et al. (2016)	Clinical	60	Sewage	6	32.8	6	+	3
Litt and Jaroni (2017)	Clinical, cattle feces	54	Cattle	7	89.7	0	+	1
Romero-Suarez et al. (2012)	Walnut	16	Walnut	26	71.6	12.6	+	2
Wang et al. (2015)	Cattle, human	41	Cattle feces	11	20.2	23.8	+	5
Murphy et al. (2013)	Dairy	20	Dairy	24	31.5	37.3	+	4
Sajben-Nagy et al. (2012)	Laboratory, mushroom	34	Mushroom	16	39.3	12.8	+	3
This work (Figure 3B)	Dairy	44	Livestock feces	26	30.2	15.2	+	4
Vu et al. (2019)	Vegetable, seafood, livestock	31	Prophage	39	21.6	11.6	+	4
This work (Figure 3A)	Dairy, laboratory	56	Fecal	26	45.6	17.4	+	4
Petsong et al. (2019)	Livestock	47	Livestock	36	14.3	10.2	+	5
Jäckel et al. (2017)	Laboratory	113	Prophage	19	6.2	11.6	+	7
Brady et al. (2017)	Beehive	40	Beehive	57	40.7	7.7	+	3
Gencay et al. (2019)	Pork meat	72	Prophage	41	22.1	13.7	+	5
Korf et al. (2019)	Clinical, poultry	64	Poultry, sewage	50	18.7	18.2	+	6
Mathieu et al. (2020)	Feces	75	Feces	166	6.1	2.8	+	5

*^*a*^Temperature measured by BMN.*

*^*b*^Nested (+) implies p < 0.05.*

**FIGURE 5:**
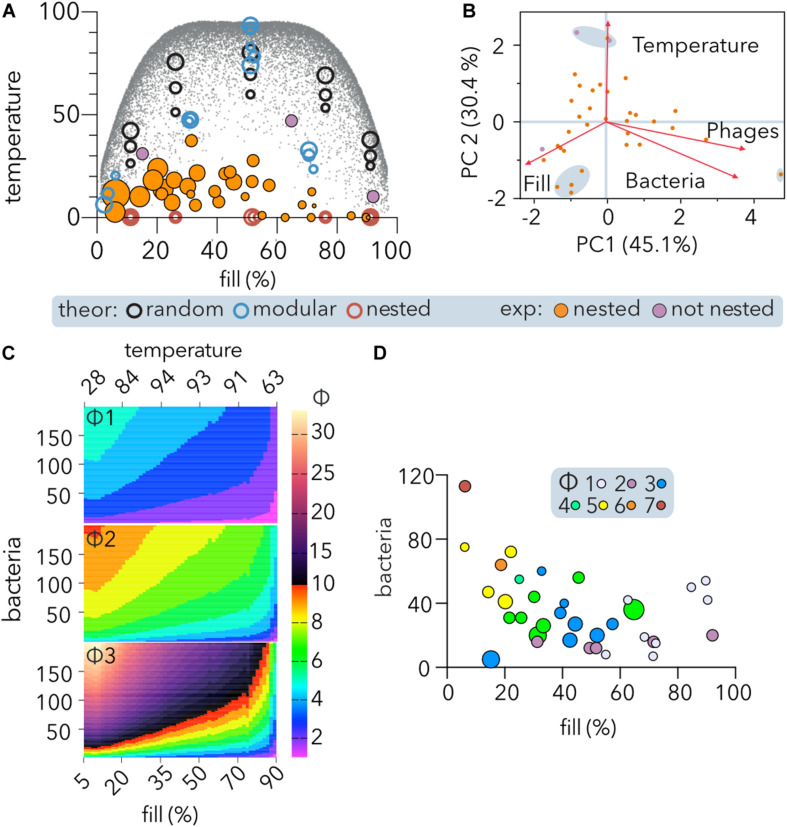
Nestedness of experimental PBINs and determination of phage cocktail sizes (Φ). **(A)** Temperature vs. fill of host range matrices nested using BMN. Experimental and theoretical datasets were compared. Each point represents one matrix, and its area reflects the matrix size. Significantly (*p* < 0.05) nested (orange) and non-significantly nested (purple) experimental matrices are distinguished. Three sizes of theoretical square matrices were considered: 400, 1,600, and 6,400 cells. The small gray dots represent theoretical square matrices obtained from Rodríguez-Gironés and Santamaría (2006). **(B)** Comparison of experimental PBINs by principal component analysis (PCA). Significantly (orange) and non-significantly (purple) nested matrices are shown. The loading score vectors are represented by red arrows (PC1 *p* = 0.0009; PC2 *p* = 0.0287). **(C)** Maximum values of Φ (phages per cocktail estimator). The maximum theoretical temperature (calculated by BMN) for each fill value (gray dots on **A**) was considered to estimate Φ for different size matrices. The number of bacteria was set to range between 5 and 200. Three alternative Φ were compared: Φ1 (Eq. 1, *n* = 4); Φ2: (Eq. 2, *n* = 2), and Φ3 (Eq. 1, *n* = 2) (see section “Materials and Methods”). **(D)** Dependence of Φ on nestedness, fill and matrix symmetry. The experimental datasets and Φ2 estimator were considered. The size of each point reflects the temperature of each matrix. The size of the phage cocktails is indicated by the color code.

*^*c*^Phage cocktail size estimated by Φ2 (Figure 5B).*

### Determining the Number of Phage Isolates Required for Biocontrol of Bacterial Populations

To study the relationship between a matrix structure (specifically, its size and fill) and its temperature, experimental (Table 2) and different kinds of theoretical matrices were incorporated into the analysis (Figure 5A). Whereas the temperature of perfectly nested matrices (0 degrees) is not affected by its size or fill, in random matrices, there is a positive correlation between temperature and size. Additionally, in random or modular matrices, the temperature depends on fill, peaking at intermediate fill values and declining at low or high fill values. Thus, the maximum temperature host range matrices can reach (gray dots, Figure 5A) follows a symmetric fourth degree polynomial function (not shown). It follows from the above that a direct comparison of the temperatures of two phage-host communities is meaningless unless the matrices representing them have the same size and fill. Remarkably, the distribution of temperatures showed that middle filled large matrices depicted values below 50, with a maximum value of 47.1, suggesting predominant gene-for-gene coevolution between phages and hosts.

Comparison of the experimental matrices (Table 2) by PCA showed (Figure 5B) that the first principal component (PC1), accounting for 45.1% of the variation, was similarly constituted by fill (35.6%), phages (34%), and bacteria (30.4%), and separating the largest matrix from the rest. The second principal component (PC2), accounting for 30.4% of the variation, was constituted mainly by temperature (74.7%), followed by fill (15%), separating the three matrices with higher temperature and a cluster of four perfectly nested matrices of similar size.

To develop an estimator of the phage cocktail size (Φ), it was taken into consideration that in PBINs, (1) the fill (f) directly indicates the host range of the phage population, (2) as the temperature (T) increases, phages tend to be less generalist, and (3) the number of bacteria (b) determines the target for biocontrol. Thus, we compared several metrics that increase with b and T and decrease with f in non-linear relationships (Figure 5C). From the alternatives, we choose a metric (Φ2 in Figure 5C) that generates cocktails between 1 and 10 for host range matrices comprising up to 200 bacteria:

$$\Phi =\left[l\,o\,g_{2}\,\left(\frac{b\cdot T}{f}+2\right)\right]$$

When considering the experimental PBINs in the meta-analysis (Table 2), this metric yielded phage cocktail sizes varying from 1 to 7 (Figure 5D). The experimental PBINs specifically built for this study (Figure 3), despite their size difference (matrix A comprises 27% more bacterial strains than matrix B and 69% more than matrix C), generated phage cocktails of the same size due to the inverse correlation of the size with the nestedness temperature. Remarkably, the experimental PBIN with the highest temperature (last row in Table 2) would require a cocktail larger than the number of phages tested, suggesting that to perform effective biocontrol of the bacterial population, additional phage strains should be isolated. Considering that this PBIN is not significantly nested and the diversity of the bacterial hosts (36 strains of 11 different *Brucella spp.)* in the matrix (Hammerl et al., 2016), this result is not unexpected.

### Hierarchical Clustering vs. Estimation of the Phage Cocktail Size

**FIGURE 6:**
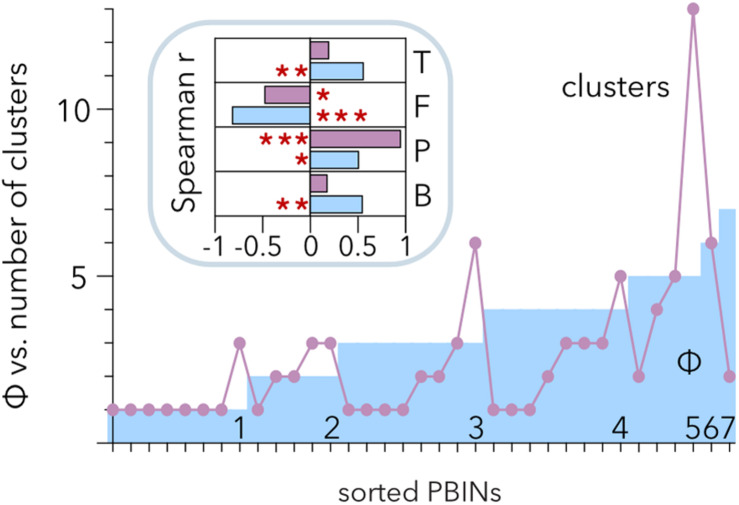
Comparison of hierarchical clustering of matrices and estimated phage cocktail size. The host range matrices (Table 2) were sorted from left to right primarily by their calculated cocktail size (Φ) and then by the number of clusters generated after applying the Ward algorithm. The inset shows the correlation of cocktail size (cyan) and the number of clusters (purple) with the temperature (T), fill (F), number of phages (P) and number of bacteria (B). The red asterisks indicate statistical significance: *p < 0.01, **p = 0.001, and ***p < 0.001.

Hierarchical clustering allows discrimination of phages with similar host ranges even if there is noise between the groups (Strauss and von Maltitz, 2017). Therefore, Ward’s algorithm poses an alternative to the analysis of Qb (Beckett and Williams, 2013) to study the structure of PBINs. Hierarchical clustering of the host range matrices (Table 2) gave rise to a distribution of clusters per matrix ranging from 1 to 13 (Figure 6). Comparison of the number of clusters and the estimation of cocktail size (Φ) showed a positive correlation between them (*r* = 0.512, *p* = 0.0017). Approximately half (49%) of the PBINs yielded higher Φ values than the number of clusters, 34% produced more clusters than the estimated cocktail size, and for the remaining 17%, the values were the same. Whereas Φ correlated significantly with all the components (temperature, fill, number of phages and number of bacteria) of the PBINs (Figure 6, inset), the number of clusters generated by Ward’s algorithm correlated significantly with only the number of phages and fill. Moreover, the strongest and most significant correlations corresponded to fill for Φ and to the number of phages for Ward’s clustering, respectively. These results indicate that the nestedness of the host range matrices cannot be detected by using this hierarchical clustering approach.

### Formulation and Evaluation of a Cocktail for Biocontrol of *E. coli*

**FIGURE 7:**
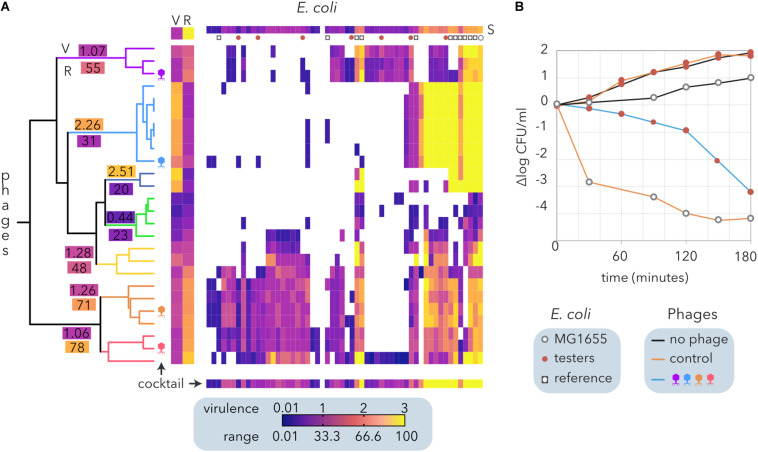
Design and evaluation of a phage cocktail for biocontrol of *E. coli* isolated from small ruminant raw milk cheeses. **(A)** Hierarchical clustering of an *E. coli* and coliphage matrix (Figure 2B) by Ward’s method. Each phage-host interaction was analyzed several times (6 > *N* > 2), and the average virulence (growth inhibition) is represented by the clearing intensity (0 = no lysis, 3 = complete lysis) of cross-streaks (Molina et al., 2020). The host range (R) and the average virulence (V) for each phage and the average susceptibility of each host strain to phages are shown (S). The clusters of phages are indicated by branch coloring in the dendrogram. The average virulence (V) and host range (R) values are shown for each cluster. The phages selected for the cocktail are schematically depicted. The expected virulence of the cocktail for each bacterial strain is shown at the bottom. Reference *E. coli* and the strains selected to test the phage cocktail are represented as indicated in the legend. **(B)** Evaluation of growth inhibition by the phage cocktails. The reference *E. coli* strain MG1655 and seven cheese-isolated *E. coli* strains with different proteomic (Molina et al., 2020) and phage sensitivity profiles **(A)** were used as controls. Two cocktails of phages are compared: the control (λ, T4, T6, and P1) and that designed from ewe feces isolates **(A)**.

In contrast to the algorithms that determine modularity and nestedness (Figure 2B), hierarchical clustering does not require bipartite matrices and allows us to consider virulence apart from the host range of the phages. To formulate a phage cocktail, we used a quantitative host range matrix comprising *E. coli* from different sources (Table 2) and coliphages isolated from sheep feces (Molina et al., 2020) that includes the same bacteria and phages depicted in Figure 3A. The hierarchical clustering of the coliphages by Ward’s method generated seven clusters (Figure 7A), each exhibiting a different host range (R) and virulence (V). Interestingly, there was a weak negative correlation between phage virulence and host range (*r* = −0.25). Noticeably, most reference *E. coli* strains exhibited higher susceptibility (S) than cheese isolates to the phages, but only one of the latter was resistant to all the phages tested.

Following the calculated Φ (Table 2), we designed a cocktail made of four phages. To this end, the clusters with the narrowest host range (<30%) were discarded, and the remaining clusters were sorted by decreasing range (not shown). Starting with the cluster with the broadest host range, combinations of four phages comprising only one phage per cluster were compared until the broadest range and highest virulence were reached (bottom line on Figure 7A). The phage cocktail was tested by inoculation of the selected phages into a combination of seven *E. coli* isolates. Host strains were selected according to their distinct proteomic profiles, which show a moderate positive correlation with their sensitivity to the phages (Molina et al., 2020). The assays were performed on reconstituted milk (Figure 7B) and LB medium (not shown), producing similar results. Whereas the proliferation of cheese-isolated *E. coli* strains was not inhibited by the control cocktail of phages (λ, T4, T6, and P1), the cocktail designed using ewe feces-isolated coliphages (Figure 7A) reduced *E. coli* counts by five logarithms after 3 h of incubation (Figure 7B). This decrease was equivalent to that observed when MG155 was inoculated with the control phage cocktail. Despite being temperate, λ phage was included in the control cocktail because it represents a paradigm amongst phages (Chatterjee and Rothenberg, 2012) and its adsorption to cheese-isolated *E. coli* strains has been previously investigated (Molina et al., 2020).

## Discussion

The use of phages to treat bacterial infections (phage therapy) or contaminations (biocontrol), notwithstanding its increasing popularity, may have some unintended side effects. Phages, despite their lethality for individual host cells, can confer an evolutionary benefit to bacterial populations (Williams, 2012). On long timescales, virulent phages may actually increase the bacterial growth rate by aiding the selection of faster-growing strains. Moreover, phages could conceivably transfer genes that augment the fitness of host strains, such as antibiotic resistance genes (Haaber et al., 2016; Gunathilaka et al., 2017). On the other hand, phage therapy entails advantages, such as low toxicity for animals and plants, high host specificity, and exponential growth of phages, which amplifies the treatment (Curtright and Abedon, 2011). To successfully control bacterial proliferation, phage cocktails are applied unless a single phage isolate infects every bacterial lineage, which is rarely the case. Therefore, the formulation of a phage cocktail constitutes a tradeoff between achieving a high reduction in the bacterial load and minimizing the side effects associated with increased complexity.

Presence-absence binary matrices are extensively used to evaluate habitat fragmentation in ecological networks, and consequently, several matrix-sorting algorithms have been developed (Almeida-Neto et al., 2008). Additionally, the coevolution of phages and bacteria has been studied by comparing the modularity and nestedness of PBINs (Beckett and Williams, 2013; Weitz et al., 2013). However, to the best of our knowledge, host range matrices have not been previously transformed into PBINs to design phage cocktails. In the meta-analysis presented here, apart from the diversity of sources, we have incorporated data including bacteria from diverse families and genera, such as *Bacillus*, *Brucella*, *Campylobacter*, coliforms, *Dickeya*, *Lactococcus*, *Listeria*, *Ochrobactrum*, *Paenibacillus*, *Pseudomonas*, *Rhodococcus*, *Salmonella*, *Staphylococcus*, and *Xanthomonas*.

The algorithm used for ordering hosts and phages in a matrix format might hinder or reveal meaningful biological information, and the nestedness could be overlooked if no packing algorithm is applied. The nestedness temperature of bipartite PBINs has been previously calculated with heuristic algorithms, such as the NTC (Poullain et al., 2008; Flores et al., 2011; Weitz et al., 2013). However, in the NTC algorithm, (1) the line of perfect order (isocline) is not uniquely defined, (2) row and column sorting does not generate a packed matrix with the lowest temperature, and (3) the null model used to determine the probabilities of finding random matrices with the same or lower temperature is not adequate for most applications (Atmar and Paterson, 1995). Our work is based on BMN (Rodríguez-Gironés and Santamaría, 2006), which implements a line of perfect order that is uniquely defined and uses genetic algorithms to determine the reordering of rows and columns that minimizes the nestedness temperature.

Our findings support that most PBINs possess a nested structure (Flores et al., 2011), indicating that difficult-to-infect hosts are infected by generalist phages (and not specialist phages). In ecology, modularity is regarded as an important feature for the maintenance of biodiversity. Likewise, nestedness is predicted to affect important properties of communities, such as stability and extinction potential. Interestingly, local adaptation may form nested patterns within overall modular networks where genetic differences between populations may limit the exchange of viruses between groups (Beckett and Williams, 2013). However, the comparison of coliphages with hosts from different sources (Figure 3A vs. Figure 3C) evinces that phage cocktails can be designed against multiple bacterial genera and suggests that nestedness prevails over modularity, indicating gene-for-gene coevolution even for relatively distant lineages. The remarkable diversity within *E*. *coli* correlates with a broad set of functions for adapting to many different environments (Hendrickson, 2009; Lukjancenko et al., 2010). Furthermore, the overlap in gene content with related species suggests a continuum rather than sharp species borders between *Shigella* spp. and *E*. *coli*. The niche expansion of *E. coli* might imply diversifying selection for coliphages and contribute to explaining the diversity of species in which they can propagate.

The estimator of phage cocktail size, Φ, does not intend to achieve the minimum possible cocktail size but to consider ecological and evolutionary information provided by the structure of PBINs that might contribute to determining the effectiveness of the cocktails. For instance, for biocontrol of the *E. coli* strains studied here, a cocktail of three phages could be designed (data not shown) that would expectedly result in the same host range but lower virulence than those of the 4-phage cocktail assayed. Similarly, to design the smallest possible phage cocktails, host range matrices could be resized by deleting “duplicated” rows and columns, i.e., phages or bacteria with the same infection pattern, but this purge would entail losing relevant information regarding the fill, nestedness temperature and modularity. Since, the cocktail formulated here was intended for the biocontrol of cheese-isolated *E. coli*, we are currently developing a challenge test in pilot plants to further test its applicability under cheese manufacturing conditions. Although the complex microbiota of cheese and the acidification might modify the effectiveness of the cocktail, our preliminary results (data not shown) indicate a reduction in *E. coli* counts during fermentation preventing cheese spoilage by early blowing. Nevertheless, industrial scale treatments could require the reformulation of phage cocktails to maintain efficacy.

The frequent use of binary host range matrices inevitably loses information and introduces bias that accentuates some features and masks others (Beckett and Williams, 2013). Conversely, the analysis of weighted phage-bacteria networks rather than just bipartite PBINs allows us to distinguish host range from virulence. Moreover, their negative correlation, which in turn might indicate that phages face life history tradeoffs (De Paepe and Taddei, 2006; Keen, 2014) such as maximizing virulence and preserving host populations for long-term exploitation, could not have been detected (Molina et al., 2020).

A long-term coevolution study in a natural community (Laanto et al., 2017) showed that phages acquired a broader host range over time and bacteria were relatively more resistant to phages from previous time points but relatively less resistant to phages from future time points. Hence, phage cocktails might require different formulations when long-term biocontrol is needed. Additionally, highly dynamic environments, such as virome-microbiome interactions in the gut (Sutton and Hill, 2019), could necessitate time-structured treatments or cycling of cocktails. The current work aimed to elucidate the relevant properties of PBINs for designing phage cocktails. A remaining challenge is to develop tools for analyzing their long-term effectiveness and smoothly integrating empirical and theoretical information.

## Materials and Methods

### Bacterial Strains and Bacteriophages

Reference bacterial strains are listed in Table 1. Most coliform strains were analyzed as described elsewhere (Molina et al., 2015). The *E. coli* K-12 strains belong to our laboratory collection (Molina et al., 1998). A total of 44 *E. coli* strains corresponding to 13 different sodium dodecyl sulfate-polyacrylamide gel electrophoresis (SDS-PAGE) protein profiles were isolated from three cheese varieties (semihard goat, soft ewe, and semihard ewe) at different ripening stages (Molina et al., 2020). All bacterial strains were grown at 37°C in lysogeny broth medium. A total of 88 coliphages and 14 *Citrobacter* phages were isolated from ewe feces and sewage, respectively. Turbid plaque phage isolates were discarded, and the remaining 26 coliphages and 5 *Citrobacter* phages were used to perform the infection analysis. Four reference coliphages (λ, T4, T6, and P1) were used from our laboratory collection to constitute the control phage cocktail (Figure 7B).

### Cheese Sampling and Isolation, Identification, and Characterization of *E. coli*

Two batches of soft ewe cheese (Torta del Casar PDO), semihard goat cheese (Ibores Cheese PDO) and semihard ewe cheese were manufactured by different local producers following traditional methods as described elsewhere (Molina et al., 2020). From each of the batches, samples of milk, curd, and 1-, 2-, 4-, and 8-week-old cheese were taken. *E. coli* was isolated by plating on chromogenic medium (Pronadisa, Spain). Identification was performed with the aid of the EnteroPluri-Test System (Liofilchem^®^, Italy) and the Biolog Microbial ID System (Biolog, United States). Strain characterization was performed by one-dimensional SDS-PAGE of whole-cell proteins. Protein samples were prepared according to Jackman (1988) and analyzed by 10% SDS-PAGE by the method of (Laemmli, 1970).

### Isolation of Bacteriophages

Coliphages were purified from sheep feces collected from local farms and treated following an enriching method (Jones and Johns, 2009) consisting of mixing 25 g of sample in 100 mL of phage suspension buffer [1% 1 M MgSO_4_ and 0.5 M CaCl_2_ (v/v)]. After 2 h of incubation (8 strokes/s) at room temperature in a stomacher VWR LB 400 (Pensilvania, United States), samples were filtered and centrifuged at 8,000 × g for 10 min. A few drops of trichloromethane were added, and the samples were shaken for 15 min at 37°C and centrifuged again at 8,000 × g for 10 min. The supernatant was filtered through a 0.22 μm pore diameter filter (MF; Millipore). *Citrobacter spp.* phages were isolated from 200 mL sewage water samples collected from the wastewater treatment plant Rincón de Caya (Badajoz, Spain). Each sample was homogenized by agitation with a Nahita blue (Beriaáin, Spain) magnetic stirrer (100 rpm, 15 min at 37°C), divided into 10 mL aliquots, centrifuged and filtered as described above. All the samples were stored at 4°C with trichloromethane to kill the remaining bacteria. Samples were screened for phages through spot assay as described elsewhere (Mirzaei and Nilsson, 2015). To detect the presence of bacteriophages in the supernatants, the bacterial hosts were sown using the double layer method. Plates were divided into 16 sectors, and aliquots of 20 μL of phage supernatant were dropped in each sector. Once dried, plates were incubated at 37°C for 18 h to let lytic zones appear. Phage strains were later purified by puncturing previously obtained lytic areas with an inoculum loop and washing it in phage suspension buffer. These suspensions were used to lyse cultures of the bacterium used in the drop test. Lysates were serially diluted and sown with the double layer method along with the corresponding bacterium. This process was repeated successively until all plaques obtained were homogeneous for at least three consecutive times.

### Host Range Determination of Coliphages and Citrobacter Phages

To identify the most effective and virulent phages, the plaque size and morphology were analyzed using indicator strains of *E. coli* (ATCC 700926) and *Citrobacter youngae* (CECT 5335). The phages producing turbid plaques, which might evince temperate phages, were discarded. To evaluate bacterial growth inhibition, cross-streak tests were carried out as detailed elsewhere (Miller, 1998). Briefly, the virulent phages were plated in nutrient agar following parallel streaks across the plate. Once dry, bacteria were plated perpendicular to phage streaks. After overnight incubation at 37°C, a picture of each plate was digitalized using a colony counter (Scan 500, Interscience). Zones of bacterial lysis were assessed with a scaling system, where 0 indicated no infection and 3 indicated a fully or nearly fully degraded bacterial lawn. Each infection assay was performed at least three times (6 > *N* > 2), and the average values were converted into an unsorted host range matrix (Molina et al., 2020) represented as a heat map (Figure 2B).

### Modularity and Hierarchical Clustering of Host Range Matrices

To estimate the modularity of the coliphages and cheese-isolated *E. coli* strains, the quantitative host range matrix (Figure 2B) was transformed into a binary form. Later, the package BiWeb (see http://github.com/tpoisot/BiWeb.), which uses the LP-BRIM sorting algorithm to find the partition that best maximizes Barber’s modularity (Qb) (Barber, 2007), was used to find groupings of highly interconnected phages and bacteria.

To determine clusters of phages sharing similar host ranges, agglomerative hierarchical clustering was performed using Ward’s method, which minimizes the total within-cluster variation. The number of clusters was established following the elbow method using R^1^.

### Nestedness of Host Range Matrices and Estimation of Cocktail Sizes

Both our data and previously published results (Table 2) were processed in the form of binary matrices, reducing the lytic spectrum to either lytic or non-lytic interactions. To quantify the nestedness and estimate the matrix temperature, five steps were carried out: (1) computing the isocline of perfect order, (2) reorganization of the matrix to maximize its nestedness by permuting rows and columns, (3) removal of multiple empty and all-presence rows and columns, (4) calculation of the matrix fill, and (5) computation of temperature by scoring distances to isocline (with each absence above the isocline and with each presence below it, there is a distance score). The temperature of the matrix is the normalized sum of distances that ranges between 0 for a perfectly nested matrix and 100.

The software used to build the nested networks was obtained from the original sources. The Nestedness Temperature Calculator (NTC) arranges the matrix by columns and then by rows, and the process is iterated eight times (Atmar and Paterson, 1995). Additionally, it provides a random null model to calculate the statistical significance of the matrix temperature. BinMatNest (BMN) (Rodríguez-Gironés and Santamaría, 2006) orders columns and rows in descending order according to their number of presence-denoting cells. Then, to pack the matrix, a genetic algorithm produces an initial set of possible solutions that are improved by the production of new variants (1,000 null matrices) with selection of the best-performing ones. This process is iterated for 2,000 generations, and the best-performing solution is used to calculate the temperature of the input matrix.

To determine the best estimator of phage cocktail size (Φ), it was considered that (1) the maximum nestedness temperature and the fill of host range matrices are strongly (*R*^2^ = 0.9995) correlated by a symmetric quartic polynomial, (2) the fill (f) indicates the host range of the phage population, (3) the temperature (T) correlates inversely with the degree of nestedness, (4) the number of bacteria (b) determines the target for biocontrol, and (5) complex cocktails (>10) increase the risk of side effects. Several functions that increase with b and T and decrease with f in non-linear relationships were evaluated (data not shown), and two were found to produce the best results:

(1) $$\Phi =\left[\sqrt[{n}]{\left(\frac{b\cdot T}{f}+n\right)}\right]$$

and

(2) $$\Phi =\left[l\,o\,g_{n}\,\left(\frac{b\cdot T}{f}+n\right)\right]$$

where n is any positive integer.

### Statistical Analysis

Principal component analysis (PCA) was carried out by estimating the principal components using the restricted maximum likelihood (REML) method. The Bartlett test was used to determine the variance in eigenvalues by calculating the Chi-square (ChiSq), degrees of freedom (DF), and the p-value (prob > ChiSq).

Two-tailed (95% confidence interval) non-parametric Spearman correlation analysis was used to evaluate the relationships between different pairs of variables throughout the text: bacterial load reduction and phage cocktail size; clusters, Φ and PBIN properties (temperature, fill, phages and bacteria); and virulence and host range of the coliphages.

### Phage Cocktail Evaluation

Phage cocktails were generated by mixing 100 μL of the selected phages after dilution of each to a final titer of 10^7^ plaque-forming units (pfu/mL) measured on the control strain *E. coli* MG1655. Bacterial cocktails were generated by mixing 1 mL of exponentially growing (optical density (O.D.) at 600 nm = 0.12) cultures grown in LB medium. Three hundred microliters of the bacterial cocktails were inoculated with phage cocktails (estimated multiplicity of infection (M.O.I.) of 10) and, after 5 min of preadsorption, were transferred to a flask with 10 mL of sterilized, reconstituted dried milk (10% w/v); the mixtures were incubated in a water bath at 37°C. Samples (50 μL) were collected at 30 min intervals for 3 h after infection, plated in nutrient agar and incubated at 37°C overnight. Colony-forming units (CFU) were counted the next day. The same experiment was carried out using LB instead of reconstituted milk, and the O.D. (600 nm) was measured every 30 min after infection for a total of 3 h (data not shown).

## Data Availability Statement

The raw data supporting the conclusions of this article will be made available by the authors, without undue reservation.

## Author Contributions

FM and JR conceived and designed the experiments. AS and RT performed the experiments. FM, MR, and AS analyzed the data. RT and IR provided bacterial strains and cheese samples. FM wrote the manuscript. All authors read and approved the final manuscript.

## Conflict of Interest

The authors declare that the research was conducted in the absence of any commercial or financial relationships that could be construed as a potential conflict of interest.

## Footnotes

1 https://uc-r.github.io/hc_clustering
